# Hospitalizations from covid-19: a health planning tool

**DOI:** 10.11606/s1518-8787.2022056004315

**Published:** 2022-06-07

**Authors:** Miguel Santolino, Manuela Alcañiz, Catalina Bolancé

**Affiliations:** I Universitat de Barcelona Institut de Recerca en Economia Aplicada Research Group on Risk in Insurance and Finance Barcelona España Universitat de Barcelona. Institut de Recerca en Economia Aplicada. Research Group on Risk in Insurance and Finance. Barcelona, España

**Keywords:** COVID-19, complications, Hospitalization, Length of Stay, Admitting Department, Hospital, Immunization, Regression Analysis

## Abstract

**OBJECTIVE:**

Estimate the future number of hospitalizations from Covid-19 based on the number of diagnosed positive cases.

**METHOD:**

Using the covid-19 Panel data recorded in Spain at the *Red Nacional de Vigilancia Epidemiológica*, Renave (Epidemiological Surveillance Network), a regression model with multiplicative structure is adjusted to explain and predict the number of hospitalizations from the lagged series of positive cases diagnosed from May 11, 2020 to September 20, 2021. The effect of the time elapsed since the vaccination program starting on the number of hospitalizations is reviewed.

**RESULTS:**

Nine days is the number of lags in the positive cases series with greatest explanatory power on the number of hospitalizations. The variability of the number of hospitalizations explained by the model is high (adjusted R^2^: 96.6%). Before the vaccination program starting, the expected number of hospitalizations on day *t* was 20.2% of the positive cases on day *t*-9 raised to 0.906. After the vaccination program started, this percentage was reduced by 0.3% a day. Using the same model, we find that in the first pandemic wave the number of positive cases was more than six times that reported on official records.

**CONCLUSIONS:**

Starting from the covid-19 cases detected up to a given date, the proposed model allows estimating the number of hospitalizations nine days in advance. Thus, it is a useful tool for forecasting the hospital pressure that health systems shall bear as a consequence of the disease.

## INTRODUCTION

The disease known as covid-19, caused by the SARS-CoV-2 virus, was declared a global pandemic by the World Health Organization in March 2020. It gave rise to an unprecedented health and social challenge^1^. The many pandemic waves have put the hospital system under stress, as it shall meet the new demand generated and, at the same time, maintain the care to patients with processes entailing from other pathologies^2^.

Several articles have focused on modeling the development of the number of new coronavirus infections, helping to understand the relationship between diagnosed cases and hospitalizations^3^. Some studies analyze the probability of hospitalization based on risk factors such as SARS-CoV-2 variant, age, sex or pre-existing diseases^6^. However, there are few studies that attempt to predict the future number of hospitalizations based on the number of newly infected cases detected, and the time elapsing between infection and hospitalization.

Nguyen et al.^8^ apply a model to investigate the short-term multivariate association between the number of hospital beds occupied and the local incidence of SARS-CoV-2 infections in the metropolitan area of Charlotte, United States. López-Izquierdo et al.^2^analyze the association between the percentage of individuals with a positive PCR, and the number of hospitalizations for SARS-CoV-2 infection, using the Poisson regression model. They focus on the analysis of the relative risk of the number of daily admissions for every 1% or 5% daily increase of new positive PCR recorded in the previous ten days (lags from 0 to 10 days).

This work, thus, aims at estimating the future number of daily hospitalizations from covid-19 based on the number of positive cases detected. Conversely to López-Izquierdo et al.^2^, we propose a simple model relating the lags in the number of positive cases with greater explanatory capacity and the number of hospitalizations. This way, there is a tool to assist in hospital planning.

For that, a regression model with a multiplicative structure is used. Specifically, the impact of the number of positive cases on hospitalizations during the 20 days following the onset of symptoms is reviewed, as most hospitalizations occur during the first 14 days after laboratory confirmation of covid-19^6^. The lag that presents greatest explanatory and predictive capacity is selected to construct the model, and the effect on the expected number of hospitalizations during time elapsed since the start of the vaccination program, December 27, 2020, is investigated. Finally, the ability of the proposed model to predict the number of hospitalizations in the fifth pandemic wave is evaluated. The selected model allows us to estimate the number of positive cases in the first pandemic wave, when the capacity of diagnosing positive results was reduced, based on cases hospitalized from covid-19 in that period.

## METHODS

### Data

Data used in this study were extracted from the covid-19 Panel. This panel is built based on the declaration of positive cases registered in Spain to the *Red Nacional de Vigilancia Epidemiológica* (Renave) by the SiViEs platform (*Sistema de Vigilancia de España*, or Spain Surveillance System), which is managed by the *Centro Nacional de Epidemiología* (National Epidemiology Center). Specifically, two databases are used. The number of positive cases is obtained from the database containing the number of cases detected through diagnostic technique and *Comunidad Autónoma* (Autonomous Community) of residence (file *casos_tecnica_ccaa.csv*). The number of hospitalizations is extracted from the database that also contains the number of admissions to intensive care units, and the number of deaths by sex, age and province of residence (file *casos_hosp_uci_def_sexo_edad_provres.csv*).

### Time Period

The time period of the series comprises from January 1, 2020 to September 20, 2021 (latest data available). It should be noted that on May 10, 2020 the criteria for counting positive cases and recording the start date were changed. Until then, the series included cases detected through a positive diagnostic test for active infection, as well as all hospitalized cases, cases admitted to intensive care units, and deaths. As of May 11, cases confirmed through PCR or antigen testing were included. On the other hand, the date of imputation of positive cases for patients with symptoms was recorded as the date of symptoms onset or, alternatively, the date of diagnosis minus six days (if registration was until May 10, 2020) or minus three days (from May 11 onward). For asymptomatic patients, the date of imputation always coincided with the date of diagnosis. Due to this change on the criteria for counting the number of positive cases detected, the analysis excluded the period prior to May 10, 2020. Thus, the period selected in this study runs from May 11, 2020 to September 20, 2021.

### Model

To model the relationship between the number of covid-19 positive cases and the number of hospitalizations for the same disease, the multiplicative structure regression model is proposed: $y_{t}\;=\;e^{\beta_{1}\;}\cdot \;e^{\beta_{2}\cdot days\_vac_{t-n}}\cdot {\chi_{t\_n}}^{\beta_{3}}\;\cdot \;e^{\beta_{t}}$ , with y_t_ being the number of hospitalizations at time *t* (*t*=1,…,*T*, with *T* equal to the total number of observations in the series, i.e. 489 days), *x*_*t*–*n*_ the number of positive cases at time *t-n*, where *n* is the number of lags (*n*=0,1,2,…,*t-1*), and *ε*_*t*_ the error term which follows a normal distribution with null expectation and standard deviation *σ.* The expression *days*_*vac*_*t*–*n*_ takes the number of days elapsed between *t-n* timepoint and the day on which the vaccination period started, December 27, 2020, taking value zero if *t-n* timepoint is prior to this date. The parameters to be estimated are *β*_1_, *β*_2_ and *β*_3_. The multiplicative structure is widely used to study the long-term relationship between time series. By applying logarithms to both sides of the expression, the following linear relationship between the number of positive results and the number of hospitalizations is obtained:


$$\log\left(y_{t}\right)\;=\;\beta_{1}\;+\;\beta_{2}\;days\_vac_{t\_n}\;+\;\beta_{3}\log\left(\chi_{t\_n}\right)\;+\;\varepsilon_{t}$$

whose parameters may be estimated by ordinary least squares.

The analysis is based on the econometric methodology for the treatment of time series^9^. First, it is investigated whether there is a seasonal component in the series associated with the day of the week (regular variations). The multiplicative model is considered for the correction of the seasonal component, i.e., it is assumed that a series may be represented as the product of three components that reflect the trend, the seasonality and error. The seasonal component is corrected by dividing the values observed by the factors associated with seasonality. Unit root tests of the series and cointegration tests are further performed to determine the number of lags to be considered in model (1). Finally, the regression model is estimated, using the variance and covariance matrix estimator that is consistent with the heteroscedasticity and autocorrelation of residuals.

## RESULTS

All calculations were performed in R 4.1.1.^10^ A first descriptive data analysis shows that the mean number of positive cases and hospitalizations differs according to the day of the week. Specifically, there is a seasonal component in which Saturdays and Sundays systematically register lower values than the other days for both series. The estimated seasonality coefficients for each day of the week are: Mon: 1.097; Tue: 1.134; Wed: 1.083; Thu: 1.025; Fri: 1.081; Sat: 0.841; Sun: 0.738, for positive cases; and Mon: 1.118; Tue: 1.069; Wed: 1.055; Thu: 1.050; Fri: 1.087; Sat: 0.832; Sun: 0.789, for the number of hospitalizations. The seasonal component is corrected by dividing the observed values of the series by the seasonal coefficients, depending on the day of the week.

**Figure 1:**
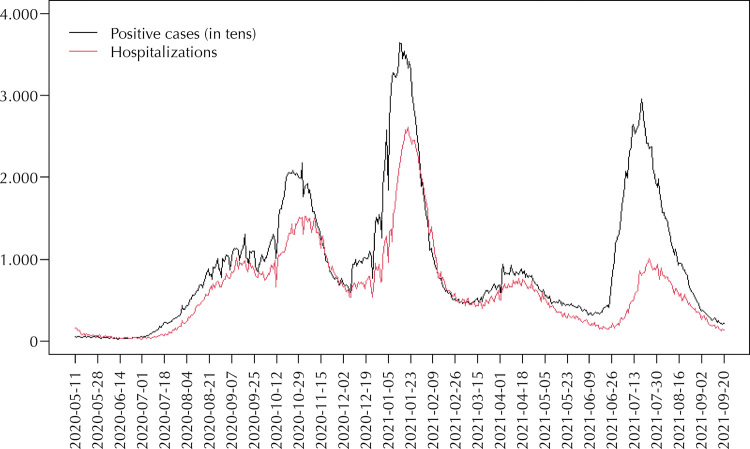
Daily series of the number of positive cases diagnosed, and the number of hospitalizations.

Once the seasonal component is corrected, the number of lags in the number of positive cases is selected. Figure 1 shows the time series of the number of positive cases (in tens), and the number of hospitalizations for the period under investigation. The behavior of both series is similar, although there is a certain lag in the number of positive cases in relation to the number of hospitalizations (translation). The seasonality of the series was analyzed using the augmented Dickey-Fuller (ADF) unit root test^11^. At a 5% significance level, the null hypothesis of non-seasonality of the series was rejected for the number of positive cases (ADF= -4.54; p = 0.01) and for the number of hospitalizations (ADF= -3.56; p = 0.03). On the other hand, the null hypothesis of non-seasonality is not rejected for positive results and hospitalizations on a logarithmic scale (ADF= -2.23; p = 0.48); ADF= -1.46; p = 0.81, respectively).

**Figure 2:**
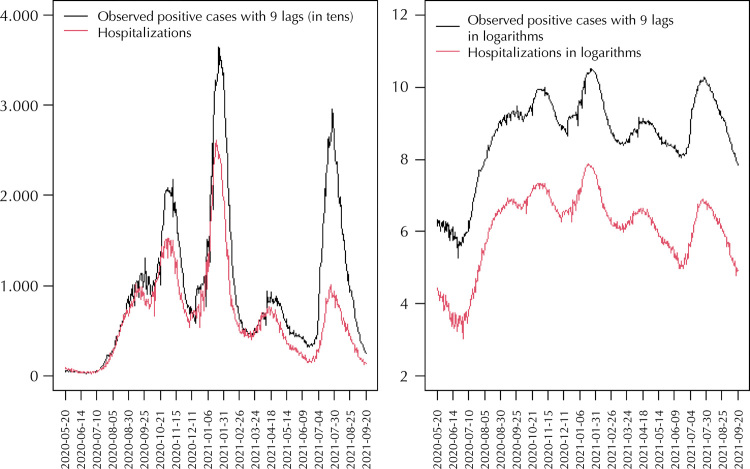
Series of the number of positive cases with 9 lags, and the number of hospitalizations (original scale and logarithmic scale).

Since the series are not seasonal on a logarithmic scale, they shall be cointegrated in order to fit the linear regression (1), and ensure that the results obtained are not spurious^9^. The cointegration of the series in logarithmic scale is tested using the Phillips-Ouliaris (PO) cointegration test, which is based on the unit root test of the residuals of the cointegrating regression^12^. The series cointegration is analyzed on a logarithmic scale for the first twenty lags of the positive cases series. Among the lagged series of positive results cointegrated with the number of hospitalizations, the one showing the greatest explanatory capacity in model (1) is selected according to the goodness-of-fit measures. Based on the results found, 9 lags in the number of positive cases are selected (PO = -116.41; p < 0.01). Figure 2 details the hospitalizations number series and the number of positive results with 9 lags on the original scale (left), and on logarithmic scale (right).

For equation (1) the error term is assumed to be homoscedastic (constant variance), and uncorrelated with lags. When these assumptions are violated, the least squares estimator of the variance-covariance matrix is inconsistent. In this study, the consistent estimator with heteroscedasticity and autocorrelation (HAC) of the variance-covariance matrix is calculated^13^. The Table presents the results of the regression model fit using estimation errors consistent with heteroscedasticity and autocorrelation.

**Table:** Fitting of the proposed regression model.

Variable	Coefficient	Standard error	p
Constant	-1.601	0.2029	< 0.001
days_vac (9 lags)	-0.003	0.0003	< 0.001
log_pos (9 lags)	0.906	0.0223	< 0.001

days_vac: days since the start of the vaccination program; log_pos: number of positive cases diagnosed (in logarithm).

Number of observations = 489.

Adjusted coefficient of determination (R^2^) = 0.9646.

The adjusted coefficient of determination is 96.46%, so it may be concluded that the explanatory power of the model is very high. The coefficients associated with the number of positive cases, and number of days elapsed since the start of vaccination are significant at 1%, with t-statistic value = 40.70 (p < 0.01) and t = -11.35 (p < 0.01), respectively. The coefficient positive sign associated with the number of positive cases suggests that the expected number of hospitalizations increases with the number of positive cases. Likewise, the coefficient negative sign associated with the number of days since the start of vaccination reflects that the number of hospitalizations decreases as the time span since the vaccination period starting increases. The residuals’ normality is not rejected at 5% with the Shapiro-Wilk test (W = 0.998; p = 0.90). At a significance level of 10%, the null hypothesis of residuals’ non-seasonality is rejected (ADF= -3.34; p = 0.06).

The estimated coefficients for model (1) are organized as follows:


$$\hat{y_{t}}=e^{-1.601}\cdot {\left(e^{-0.003}\right)}^{days\_vac_{t-n}}\cdot {\chi_{t-n}}^{0.906}\;=0.202\;\cdot \;{\left(0997\right)}^{days\_vac_{t-n}}\cdot {\chi_{t-n}}^{0.906}$$

This allows us to conclude that, before the vaccination program started, the expected number of hospitalizations on day *t* is 20.2% of the positive cases on day *t*-9 raised to 0.906. After the start of the vaccination program, the percentage of 20.2% is reduced to a rate of 0.3% a day.

### Predicted Number of Positive Cases and Hospitalizations

**Figure 3:**
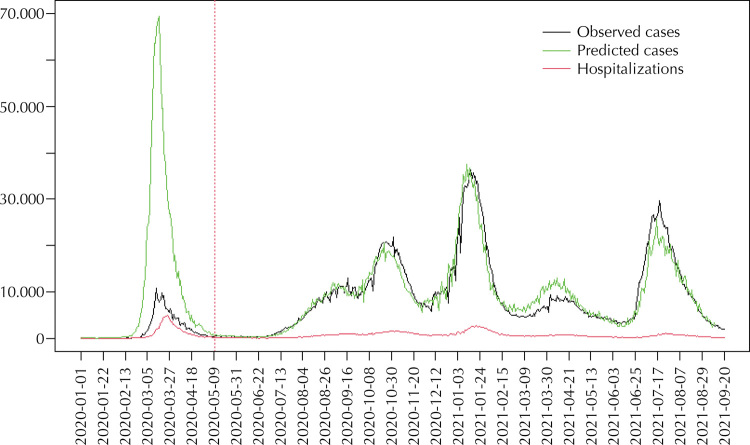
Comparison between the number of observed and predicted positive cases as a function of the number of hospitalizationsa.

^a^ The data used to fit the model are from May 11, 2020 to September 20, 2021. The number of positive cases predicted between January 1, 2020 and May 10, 2020 (first pandemic wave) are calculated based on hospitalizations observed in that period, assuming the validity of the estimated model for the subsequent period.

Information is available for the number of hospitalizations between January 1, 2020 and May 10, 2020. For the calculation of the number of hospitalizations, there was no change to the criteria at the end of that period, as was the case with the calculation of the number of positive cases. With the coefficients shown in the Table, the number of positive cases for the period January 1, 2020 to May 10, 2020 that would have predicted the number of hospitalizations we observed in that period, is calculated. Figure 3 compares the calculated number of positive cases with the number of positive cases actually recorded.

For the first pandemic wave, the number of daily positive cases that would predict the number of hospitalizations observed is much higher than that shown on the official records. The maximum number of positive cases between January 1, 2020 and May 10, 2020 would be 69,602 individuals a day, well above the recorded maximum of 10,743 individuals diagnosed positive. On more than 25% of the days in that period the computed number of positive cases exceeds the maximum value recorded.

**Figure 4:**
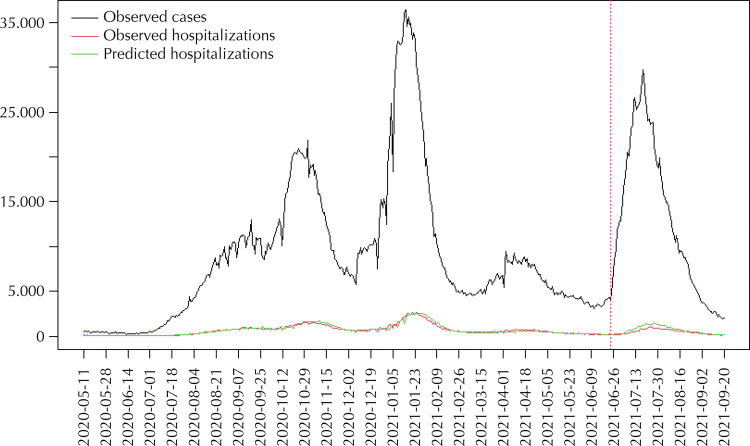
Comparison between the actual number of hospitalizations and the prediction made by the model based on observed positive casesa.

^a^ The data used for the model fit are from May 11, 2020 to June 24, 2021. Predictions on the number of hospitalizations between June 25, 2021 and September 20, 2021 (fifth pandemic wave) are made for an out-of-sample data set.

Finally, the predictive ability of the model for the fifth pandemic wave is analyzed. The fifth wave is selected to evaluate the predictive ability of the model in order to have a large number of observations on the model calibration. Model (1) is refitted for the time period from May 11, 2020 to June 24, 2021. Once the model has been estimated, the prediction of the number of hospitalizations from June 25, 2021 to September 20, 2021 (out-of-sample prediction) is performed. Figure 4 shows the prediction of the number of hospitalized cases, and compares it with the observed number. As can be seen, the model satisfactorily predicts the number of hospitalizations for that period.

## DISCUSSION

A simple way of modeling the relationship between the number of positive cases detected and the number of hospitalizations due to covid-19 is proposed. The model shows satisfactory goodness-of-fit to the data, so one may conclude that there is a multiplicative relationship between the number of cases diagnosed and the number of hospitalizations (additive between logarithmic transformations). Other studies have considered the exponential relationship between the number of hospitalizations and the percentage of positive PCR tests^2^.

For the first wave of the pandemic there was a low diagnostic capacity due to very restricted testing^14^. In that period, official records probably recorded hospitalizations from covid-19 better than the number of infections. The proposed model allows us to approximate the actual number of positive cases in the first wave based on the number of hospitalized covid-19 cases reported on official records. The results suggest that positive cases in the first wave are strongly underreported on these records. It should be noted that positive cases in the first wave computed in this article are those that would have been detected had the same diagnostic capacity been available as of May 11, 2020. In other words, the proportion of infections that remained undetected after May 11 would not be included in this estimate^3^.

The different pandemic waves gave effect to significant hospital pressure^5^ that should be managed with the available resources. The proposed model allows using the number of positive cases known to date to estimate the number of hospitalizations due to SARS-CoV-2 infection up to nine days in advance. The explanatory power and predictive behavior of the model in that time frame are very satisfactory. This makes it a useful tool for making decisions on hospital management issues. López-Izquierdo et al.^2^ suggest that the second and sixth lags of the percentage of PCR-positive confirmed results express the strongest association with the number of hospital admissions. These results would be in line with this study, which concludes that the ninth lag (nine-day difference between the onset of symptoms and hospitalization) is the one that shows the best explanatory capacity for the number of positive results, followed by the fourth lag (four-day difference).

Vaccination against covid-19 drastically reduces the risk of hospitalization^15,16^. The proposed model includes a variable that accounts for the number of days elapsed since the start of the vaccination period in Spain. Results suggest that the number of hospitalizations in relation to the number of positive cases decreases as the start of the vaccination program increases. The effect of the time from the beginning of the pandemic to the start of the vaccination period was reviewed, and the coefficient associated with the time elapsed in this interval showed no relationship with the number of hospitalizations. However, after December 27, 2020, the number of hospitalizations declines as the time elapsed from that date increases. Before then, it does not seem to be a reduction in the percentage of hospitalizations in relation to the number of positive cases diagnosed, which could mean that the virus effects severity remained constant until the emergence of the vaccine.

This work is not exempt from limitations. For example, the proposed model does not include information on the sex of individuals, although some studies suggest that being male is a risk factor of hospitalization from covid-19^17^. Unfortunately, this information is not available on the databases used. On the other hand, this work is carried out for Spain as a whole. Although the vaccination campaign began nationwide on December 27, 2020, not all autonomous communities followed the same vaccination strategy or recorded their progress in the same way. This may have led to differences that are not visible in this analysis.

The methodology proposed herein may be used as a reference to investigate the relationship between diagnosed cases and hospitalizations in other pathologies susceptible to hospitalization, especially in epidemics with waves of contagion. Based on the cases detected in primary care, the number of patients requiring hospital stay could be approximated, as well as the expected lag between diagnosis and the need to admit the patient. It should be noted that this study highlights the importance of having reliable, homogeneous and updated information for predicting the behavior of indicators of great interest in public health.
