# Supplementary material for: Hospitalizations from covid-19: a health planning tool
Source: Rev Saude Publica. 2022 Jun 7;56:51. doi: 10.11606/s1518-8787.2022056004315 (PMC9239335; doi:10.11606/s1518-8787.2022056004315)
Supplement: Translation

# Translation: Ingresos hospitalarios por covid-19: una herramienta de planificación sanitaria

**Authors:** Miguel Santolino, Manuela Alcañiz, Catalina Bolancé

**Affiliations:** I España Universitat de Barcelona. Institut de Recerca en Economia Aplicada. Research Group on Risk in Insurance and Finance. Barcelona, España

**Keywords:** COVID-19, complicaciones, Hospitalización, Tiempo de Internación, Servicio de Admisión en Hospital, Inmunización, Análisis de Regresión

## Abstract

**OBJETIVO:**

Predecir el número futuro de hospitalizaciones por covid-19 a partir del número de casos positivos diagnosticados.

**MÉTODO:**

Usando datos del Panel covid-19 registrados en España en la Red Nacional de Vigilancia Epidemiológica (Renave), se ajusta un modelo de regresión con estructura multiplicativa para explicar y predecir el número de hospitalizaciones a partir de la serie retardada de casos positivos diagnosticados durante el periodo entre el 11 de mayo de 2020 y el 20 de septiembre de 2021. Se analiza el efecto sobre el número de hospitalizaciones del tiempo transcurrido desde el inicio del programa de vacunación.

**RESULTADOS:**

El número de retardos de la serie de casos positivos que mayor capacidad explicativa tiene sobre el número de hospitalizaciones es de nueve días. La variabilidad del número de hospitalizaciones explicada por el modelo es elevada (R^2^ ajustado: 96,6%). Antes del inicio del programa de vacunación, el número esperado de ingresos hospitalarios en el día *t* era igual al 20,2% de los casos positivos del día *t*-9 elevado a 0,906. Iniciado el programa de vacunación, este porcentaje se redujo un 0,3% diario. Con el mismo modelo se obtiene que en la primera ola de la pandemia el número de casos positivos fue más de seis veces el que figura en los registros oficiales.

**CONCLUSIONES:**

Partiendo de los casos de covid-19 detectados hasta una fecha, el modelo propuesto permite estimar el número de hospitalizaciones con nueve días de antelación. Ello lo convierte en una herramienta útil para prever con cierta anticipación la presión hospitalaria que el sistema sanitario tendrá que soportar como consecuencia de la enfermedad.

## INTRODUCCIÓN

La enfermedad conocida como covid-19, causada por el virus SARS-CoV-2, fue declarada como pandemia global por la Organización Mundial de la Salud en marzo de 2020 originando un reto sociosanitario sin precedentes^1^. Las diferentes olas de la pandemia han puesto en tensión al sistema hospitalario, que debe atender a la nueva demanda generada y, al mismo tiempo, mantener la asistencia a los pacientes con procesos derivados de otras patologías^2^.

Diferentes artículos se han centrado en modelizar la evolución del número de nuevos infectados por coronavirus, ayudando a entender la relación entre los casos diagnosticados y las hospitalizaciones^3^. Algunos trabajos analizan la probabilidad de ingreso hospitalario del paciente según factores de riesgo como la variante de SARS-CoV-2, la edad, el sexo o enfermedades preexistentes^6^. Sin embargo, son escasos los estudios que tratan de predecir el número futuro de ingresos hospitalarios en función del número de casos detectados de recién infectados y del tiempo que transcurre entre la infección y el ingreso hospitalario.

Nguyen et al.^8^ aplican un modelo para analizar la asociación multivariante a corto plazo entre el número de camas ocupadas en los hospitales y la incidencia local de infecciones por SARS-CoV-2 en el área metropolitana de Charlotte, Estados Unidos. López-Izquierdo et al.^2^analizan la asociación entre el porcentaje de individuos con una PCR positiva y el número de ingresos hospitalarios por una infección por SARS-CoV-2 mediante el modelo de regresión de Poisson. Se centran en el análisis del riesgo relativo del número de ingresos diario para cada aumento de un 1% o 5% diario de las PCR positivas nuevas registradas en los diez días anteriores (retardos de 0 a 10 días).

Ante esto, este trabajo se propone estimar el número futuro de hospitalizaciones diarias por covid-19 en base al número de casos positivos detectados. A diferencia de López-Izquierdo et al.^2^, proponemos un modelo simple que relacione el retardo en el número de casos positivos con mayor capacidad explicativa y el número de hospitalizaciones. De este modo, se dispone de una herramienta que ayude en la planificación hospitalaria.

Para ello, se utiliza un modelo de regresión con estructura multiplicativa. Concretamente, se analiza el impacto del número de casos positivos sobre las hospitalizaciones durante los 20 días siguientes al inicio de los síntomas, ya que la mayor parte de los ingresos hospitalarios se producen en los primeros 14 días desde la confirmación del covid-19 en laboratorio^6^. Se selecciona el retardo que muestra una mayor capacidad explicativa y predictiva para la construcción del modelo, y se analiza el efecto sobre el número esperado de hospitalizaciones del tiempo transcurrido desde el inicio del programa de vacunación, el 27 de diciembre de 2020. Por último, se evalúa la capacidad del modelo propuesto en pronosticar el número de hospitalizaciones en la quinta ola de la pandemia. El modelo seleccionado nos permite aproximar el número de casos positivos en la primera ola de la pandemia, cuando la capacidad diagnóstica de positivos era reducida, a partir de los casos hospitalizados por covid-19 durante ese periodo.

## MÉTODOS

### Datos

Los datos utilizados en este estudio proceden del Panel covid-19 que se construye a partir de la declaración de los casos positivos registrados en España a la Red Nacional de Vigilancia Epidemiológica (Renave) por la plataforma SiViEs (Sistema de Vigilancia de España), que gestiona el Centro Nacional de Epidemiología. En concreto se utilizan dos bases de datos. El número de casos positivos se obtiene a partir de la base de datos que recoge el número de casos detectados por técnica diagnóstica y Comunidad Autónoma de residencia (fichero *casos_tecnica_ccaa.csv*). A su vez, el número de hospitalizaciones se extrae de la base de datos que contiene también el número de ingresos en unidades de cuidados intensivos y el número de defunciones por sexo, edad y provincia de residencia (fichero *casos_hosp_uci_def_sexo_edad_provres.csv*).

### Periodo Temporal

El periodo temporal de las series cubre desde el 1 de enero de 2020 hasta el 20 de septiembre de 2021 (último dato disponible). Es preciso tener en cuenta que el 10 de mayo de 2020 se produjo un cambio de criterio en el cómputo de casos positivos y en el registro de la fecha de inicio. Hasta esa fecha, la serie incluía casos detectados por una prueba diagnóstica positiva de infección activa, así como todos aquellos casos hospitalizados, ingresados en unidades de cuidados intensivos y defunciones; a partir del 11 de mayo se incluyeron casos confirmados por PCR o por pruebas de antígeno. Por otro lado, la fecha de imputación de los casos positivos para los pacientes con síntomas se consignó como la fecha en que estos se iniciaron o, en su defecto, la fecha de diagnóstico menos seis días (si el registro se hizo hasta el 10 de mayo de 2020) o menos tres días (a partir del 11 de mayo); para los asintomáticos, la fecha de imputación coincide siempre con la fecha de diagnóstico. Debido a este cambio de criterio en el cómputo de casos positivos detectados, se decidió excluir del análisis el periodo anterior al 10 de mayo de 2020. Así, el periodo seleccionado en este estudio abarca desde el 11 de mayo de 2020 hasta el 20 de septiembre de 2021.

### Modelo

Para modelizar la relación entre el número de casos positivos por covid-19 y el número de hospitalizaciones por la misma enfermedad, se propone el modelo de regresión de estructura multiplicativa $y_{t}\;=\;\;e^{\beta_{1}\;\cdot \;e\beta_{2}\;\cdot \;días\_vac_{t-n}}\;\cdot \;{\chi_{t-n}}^{\beta_{3}}\;\cdot \;e^{\beta_{t}}$ , siendo y_t_ el número de hospitalizaciones en el momento *t* (*t*=1,…,*T*, con *T* igual al número total de observaciones en la serie, es decir, 489 días), *x*_*t*–*n*_ el número de casos positivos en el momento *t-n*, donde *n* es el número de retardos (*n*=0,1,2,…,*t-1*), y *ε*_*t*_ el término de error que se distribuye según una normal con esperanza nula y desviación típica *σ.* La expresión *días*_*vac*_*t*–*n*_ recoge el número de días transcurridos entre el momento *t-n* y el día en que se inició el periodo de vacunación, el 27 de diciembre de 2020, tomando valor cero si el momento *t-n* se sitúa antes de esta fecha. Los parámetros a estimar son *β*_1_, *β*_2_ y *β*_3_. La estructura multiplicativa es ampliamente utilizada para estudiar la relación a largo plazo entre series temporales, ya que, aplicando logaritmos a ambos lados de la expresión, se obtiene la siguiente relación lineal entre el número de positivos y el número de hospitalizaciones:


$$log(y_{t})=\beta_{1}+\;\beta_{2}\;días\_vac_{t-n}+\;\beta_{3}\;log(x_{t-n})+\varepsilon_{t}$$

cuyos parámetros pueden ser estimados por mínimos cuadrados ordinarios.

El análisis se realiza a partir de metodología econométrica para el tratamiento de series temporales^9^. Primero, se investiga si existe en las series componente estacional asociada al día de la semana (variaciones regulares). Se considera el modelo multiplicativo para la corrección de la componente estacional, es decir, se asume que una serie se puede representar como el producto de tres componentes que reflejan la tendencia, la estacionalidad y el error. La corrección de la componente estacional se lleva a cabo dividiendo los valores observados entre los factores asociados a la estacionalidad. Posteriormente, se realizan las pruebas de raíces unitarias de las series y de cointegración para determinar el número de retardos que considerar en el modelo (1). Finalmente, se estima el modelo de regresión con el estimador de la matriz de varianzas y covarianzas consistente con heterocedasticidad y autocorrelación de los residuos.

## RESULTADOS

Todos los cálculos se realizan en R 4.1.1.^10^ En un primer análisis descriptivo de los datos se detecta que el número medio de casos positivos y hospitalizaciones difiere según el día de la semana. Concretamente, se aprecia una componente estacional en la que los sábados y domingos registran de forma sistemática valores más bajos que el resto de los días para ambas series. Los coeficientes de estacionalidad estimados para cada día de la semana son: Lu: 1,097; Ma: 1,134; Mi: 1,083; Ju: 1,025; Vi: 1,081; Sa: 0,841; Do: 0,738, para casos positivos; y Lu: 1,118; Ma: 1,069; Mi: 1,055; Ju: 1,050; Vi: 1,087; Sa: 0,832; Do: 0,789, para el número de hospitalizaciones. La componente estacional se corrige dividiendo los valores observados de las series entre estos coeficientes estacionales, según el día de la semana.

Una vez corregida la componente estacional, se procede a la selección del número de retardos en el número de casos positivos. En la Figura 1 se muestran las series temporales del número de casos positivos (en decenas) y el número de hospitalizaciones para el periodo analizado. El comportamiento de las dos series es similar, si bien se aprecia cierto retardo en el número de positivos respecto al número de hospitalizaciones (translación). Se analiza la estacionariedad de las series mediante la prueba de Dickey-Fuller aumentada (DFA) de raíz unitaria^11^. Con un nivel de significación del 5% se rechaza la hipótesis nula de no estacionariedad de las series para el número de casos positivos (DFA = - 4,54; p = 0,01) y para el número de hospitalizaciones (DFA = -3,56; p = 0,03). En cambio, en escala logarítmica no se rechaza la hipótesis nula de no estacionariedad para positivos ni hospitalizaciones (DFA = -2,23; p = 0,48) ; (DFA = -1,46; p = 0,81) , respectivamente.

**Figura 1:** Series diarias del número de positivos diagnosticados y del número de hospitalizaciones.

Puesto que las series no son estacionarias en escala logarítmica se requiere que estén cointegradas para poder ajustar la regresión lineal (1) y garantizar que los resultados obtenidos no sean espurios^9^. Se realiza el contraste de cointegración de las series en escala logarítmica mediante la prueba de cointegración de Phillips-Ouliaris (PO) que se basa en realizar la prueba de raíz unitaria de los residuos de la regresión de cointegración^12^. Se analiza la cointegración de las series en escala logarítmica para los veinte primeros retardos de la serie de casos positivos. Entre las series retardadas de positivos cointegradas con el número de hospitalizaciones, se selecciona aquella que muestra una mayor capacidad explicativa en el modelo (1) según las medidas de bondad del ajuste. A partir de los resultados obtenidos, se seleccionan 9 retardos en el número de casos positivos (PO = -116,41; p < 0,01). La Figura 2 detalla las series del número de hospitalizaciones y el número de positivos con 9 retardos en escala original (izquierda) y en escala logarítmica (derecha).

**Figura 2:** Series del número de casos positivos con 9 retardos y del número de hospitalizaciones (escala original y escala logarítmica).

En la ecuación (1) se asume que el término de error es homocedástico (varianza constante) y no correlacionado con los retardos. Cuando estos supuestos se violan, el estimador por mínimos cuadrados de la matriz de varianzas y covarianzas es no consistente. En este estudio se calcula el estimador consistente con heterocedasticidad y autocorrelación (HAC, según sus siglas en inglés) de la matriz de varianzas y covarianzas^13^. La Tabla presenta los resultados del ajuste del modelo de regresión con los errores de estimación consistentes con heterocedasticidad y autocorrelación.

**Tabla:** Ajuste del modelo de regresión propuesto.

Variable	Coeficiente	Error estándar	p
Constante	-1,601	0,2029	< 0,001
días_vac (9 retardos)	-0,003	0,0003	< 0,001
log_pos (9 retardos)	0,906	0,0223	< 0,001

días_vac: días desde el inicio del programa de vacunación; log_pos: número de casos positivos diagnosticados (en logaritmo).

Número de observaciones = 489.

Coeficiente de determinación ajustado (R^2^) = 0,9646.

El coeficiente de determinación ajustado es del 96,46%, por lo que se puede concluir que la capacidad explicativa del modelo es muy alta. Los coeficientes asociados al número de casos positivos y número de días transcurridos desde el inicio de la vacunación son significativos al 1%, con valor del estadístico t = 40,70 (p < 0,01) y t = -11,35 (p < 0,01), respectivamente. El signo positivo del coeficiente asociado al número de casos positivos indica que el número esperado de hospitalizaciones aumenta con el número de casos positivos. Del mismo modo, el signo negativo del coeficiente asociado al número de días transcurridos desde el inicio de la vacunación refleja que el número de ingresos hospitalarios se reduce a medida que aumenta el tiempo desde que se inició el periodo de vacunación. La normalidad de los residuos no se rechaza al 5% con la prueba de Shapiro-Wilk (W = 0,998; p = 0,90). Con un nivel de significación del 10% se rechaza la hipótesis nula de no estacionariedad de los residuos (DFA = -3,34; p = 0,06).

Los coeficientes estimados del modelo (1) se organizan de la siguiente forma:


$$\hat{y_{t}}=e^{-1,601}\cdot {\left(e^{-0,003}\right)}^{días\_vac_{t-n}}\cdot {\chi_{t-n}}^{0,906}\;=0.202\;\cdot \;{\left(0,997\right)}^{días\_vac_{t-n}}\cdot {\chi_{t-n}}^{0,906}$$

Esto nos permite concluir que, antes del inicio del programa de vacunación, el número esperado de hospitalizaciones en el día *t* es el 20,2% de los casos positivos del día *t*-9 elevado a 0,906. Después del inicio del programa de vacunación, el porcentaje del 20,2% se reduce a una tasa del 0,3% diario.

### Predicción del Número de Casos Positivos y Hospitalizaciones

Se dispone de información del número de hospitalizaciones entre el 1 de enero de 2020 y el 10 de mayo de 2020. En el cómputo del número de hospitalizaciones no hubo un cambio de criterio al final de dicho periodo, como sí sucedió con el modo de computar los casos positivos. Con los coeficientes mostrados en la Tabla, se calcula el número de casos positivos para el periodo entre el 1 de enero de 2020 y el 10 de mayo de 2020 que habría pronosticado el número de hospitalizaciones que observamos en ese periodo. En la Figura 3 se compara este número calculado de casos positivos con el número de casos positivos realmente registrados.

**Figura 3:** Comparación entre el número de casos positivos observados y pronosticados en función del número de hospitalizacionesa.

^a^ Los datos utilizados para el ajuste del modelo se sitúan entre el 11 de mayo de 2020 y el 20 de septiembre del 2021. El número de casos positivos pronosticados entre el 1 de enero de 2020 y el 10 de mayo de 2020 (primera ola de la pandemia) se calculan en base a las hospitalizaciones observadas en ese periodo, asumiendo la validez del modelo estimado para el periodo posterior.

En la primera ola de la pandemia, el número de casos positivos diarios que pronosticarían el número de hospitalizaciones observadas es muy superior al mostrado en los registros oficiales. El número máximo de casos positivos entre el 1 de enero de 2020 y el 10 de mayo de 2020 sería de 69.602 personas al día, muy por encima del máximo registrado de 10.743 personas diagnosticadas positivas. En más del 25% de los días en este periodo el número computado de casos positivos supera el valor máximo registrado.

Por último, se analiza la capacidad de predicción del modelo en la quinta ola de la pandemia. Se selecciona la quinta ola para evaluar la capacidad de predicción del modelo con el fin de disponer de un número elevado de observaciones en la calibración del modelo. Se ajusta nuevamente el modelo (1) para el periodo temporal entre el 11 de mayo de 2020 y el 24 de junio de 2021. Una vez estimado el modelo, se realiza la predicción del número de hospitalizaciones entre el 25 de junio de 2021 y el 20 de setiembre de 2021 (predicción fuera de la muestra). En la Figura 4 se muestra la predicción del número de casos hospitalizados y se compara con el número observado. Como se puede apreciar, el modelo predice de forma satisfactoria el número de hospitalizaciones para dicho periodo.

**Figura 4:** Comparación entre el número real de hospitalizaciones y la predicción realizada por el modelo en función de los casos positivos observadosa.

^a^ Los datos utilizados para el ajuste del modelo se sitúan entre el 11 de mayo de 2020 y el 24 de junio de 2021. Las predicciones en el número de hospitalizaciones realizadas entre el 25 de junio de 2021 y el 20 setiembre de 2021 (quinta ola de la pandemia) se realizan para un conjunto de datos fuera de la muestra.

## DISCUSIÓN

Se propone un modo sencillo de modelizar la relación entre el número de casos positivos detectados y el número de hospitalizaciones por covid-19. El modelo muestra un buen ajuste a los datos, por lo que se puede concluir que existe una relación multiplicativa entre el número de casos diagnosticados y el número de ingresos hospitalarios (aditiva entre las transformaciones logarítmicas). Otros trabajos han considerado la relación exponencial entre el número de hospitalizaciones y el porcentaje de positivos por PCR^2^.

En la primera ola de la pandemia hubo una baja capacidad diagnóstica al estar muy restringidas las pruebas^14^. Durante ese periodo, los registros oficiales contabilizaron probablemente mejor los ingresos hospitalarios por covid-19 que el número de contagios. El modelo propuesto permite aproximar el número real de casos positivos en la primera ola a partir de los casos hospitalizados por covid-19 que constan en los registros oficiales. Los resultados sugieren que los casos positivos en la primera ola están fuertemente infracontabilizados en dichos registros. Es necesario señalar que los casos positivos en la primera ola que se computan en este artículo son los que se habrían detectado si se hubiera dispuesto de la misma capacidad diagnóstica que a partir del 11 mayo de 2020. Es decir, la proporción de contagios que siguieron sin detectarse a partir del 11 de mayo no estaría recogida en esta estimación^3^.

Las diferentes olas de la pandemia generan una importante presión hospitalaria^5^, que es preciso gestionar con los recursos disponibles. El modelo propuesto permite usar el número de casos positivos conocidos hasta una fecha para estimar el número de ingresos hospitalarios por infección con SARS-CoV-2 con hasta nueve días de antelación. La capacidad explicativa y el comportamiento predictivo del modelo en ese lapso de tiempo son muy satisfactorios. Ello lo convierte en una herramienta útil para la toma de decisiones sobre gestión hospitalaria. López-Izquierdo et al.^2^ indican que el segundo y sexto retardos del porcentaje de positivos confirmados por PCR son los que muestran una mayor asociación con el número de ingresos hospitalarios. Estos resultados estarían en línea con este estudio, en el que se concluye que el retardo noveno (diferencia de nueve días entre la aparición de los síntomas y la hospitalización) es el que muestra mejor capacidad explicativa del número de positivos, seguido del retardo cuarto (cuatro días de diferencia).

La vacunación contra el covid-19 reduce drásticamente el riesgo de hospitalización^15,16^. En el modelo propuesto se incluye una variable que contabiliza el número de días transcurridos desde el inicio del periodo de vacunación en España. Los resultados indican que el número de ingresos hospitalarios en relación al número de casos positivos se reduce a medida que aumenta el tiempo desde que se inició el programa de vacunación. Se analizó el efecto del tiempo desde el inicio de la pandemia hasta el inicio del periodo de vacunación, y el coeficiente asociado al tiempo transcurrido en este intervalo no mostró relación con el número de hospitalizaciones. Sin embargo, a partir del 27 de diciembre de 2020, el número de hospitalizaciones decae a medida que aumenta el tiempo transcurrido desde esa fecha. Antes de esta fecha no parece producirse una reducción en el porcentaje de hospitalizaciones en relación al número de positivos diagnosticados, lo que podría significar que la gravedad de los efectos del virus se mantuvo constante hasta la aparición de la vacuna.

Este trabajo no está exento de limitaciones. Por ejemplo, el modelo propuesto no incluye información sobre el sexo de los individuos, aunque algunos estudios sugieren que ser hombre es un factor de riesgo para el ingreso hospitalario por covid-19^17^. Desafortunadamente, no se dispone de esta información en las bases de datos utilizadas. Por otro lado, este trabajo se realiza para el conjunto de España. Si bien la campaña de vacunación empezó en todo el país el día 27 de diciembre de 2020, no todas las Comunidades Autónomas siguieron la misma estrategia de vacunación ni registraron su progreso del mismo modo; ello pudo dar lugar a diferencias que no son visibles en este análisis.

La metodología propuesta en este artículo puede servir de referencia para estudiar la relación entre casos diagnosticados e ingresos hospitalarios en otras patologías susceptibles de requerir hospitalización, especialmente en epidemias que cursan con olas de contagios. A partir de los casos detectados en atención primaria, se podría aproximar el número de pacientes que requerirán estancia hospitalaria, así como el retardo esperado entre el diagnóstico y la necesidad de ingresar al paciente. Cabe señalar que este estudio resalta la importancia de disponer de información fiable, homogénea y actualizada para la realización de predicciones del comportamiento de indicadores de gran interés en salud pública.
